# TRIM26-mediated NKRF degradation drives Osimertinib resistance through SNRPD2-dependent stress granule formation in lung adenocarcinoma

**DOI:** 10.1038/s41419-026-08787-x

**Published:** 2026-04-24

**Authors:** Tao Wang, Hai-Yan Yang, Xing Wang, Xin-Hao Han, Zhen Zhang, Yu Zhang, Xiao-Jian Han, Zhuo Lu

**Affiliations:** 1 Institute of Geriatrics, Jiangxi Provincial People’s Hospital, The First Affiliated Hospital of Nanchang Medical College, Nanchang, China; 2 Centre for Medical Research and Translation, Jiangxi Provincial People’s Hospital, The First Affiliated Hospital of Nanchang Medical College, Nanchang, China; 3 Institute of Clinical Medicine, Jiangxi Provincial People’s Hospital, The First Affiliated Hospital of Nanchang Medical College, Nanchang, China; 4 Institute of Biomedical Innovation, Nanchang University, Nanchang, China; 5 China-Japan Friendship Jiangxi Hospital, National Regional Center for Respiratory Medicine, Nanchang, China; 6 Department of Thoracic Surgery, The First Affiliated Hospital, Jiangxi Medical College, Nanchang University, Nanchang, China

**Keywords:** Non-small-cell lung cancer, Ubiquitylation, Transcriptional regulatory elements

## Abstract

Osimertinib is the standard first-line therapy for EGFR-mutant lung adenocarcinoma; however, the inevitable development of acquired resistance leads to disease progression and treatment failure. While established resistance mechanisms primarily involve genetic alterations, stress-adaptive pathways, particularly stress granule–mediated therapeutic tolerance, remain poorly understood. This study aims to elucidate the transcriptional and post-translational mechanisms governing stress granule–mediated survival and their contribution to Osimertinib resistance in lung adenocarcinoma. We identify NF-κB repressing factor (NKRF) as a critical suppressor of Osimertinib resistance, whose expression is markedly reduced in resistant lung adenocarcinoma cells. Restoration of NKRF significantly sensitized resistant cells to Osimertinib in vitro and inhibited tumor growth in xenograft models. Mechanistically, NKRF directly repressed transcription of the ribonucleoprotein component Small nuclear ribonucleoprotein D2 (SNRPD2), thereby constraining stress granule formation and attenuating drug tolerance. We further demonstrate that the E3 ubiquitin ligase TRIM26 interacts with NKRF and promotes its K48-linked ubiquitination at Lys411, leading to proteasomal degradation. This process sustains SNRPD2 expression and enhances stress granule assembly. Genetic depletion of TRIM26 restored NKRF stability, suppressed stress granule formation, and re-sensitized resistant tumors to Osimertinib, effects that were abrogated by concomitant NKRF silencing. Collectively, this study defines a previously unrecognized TRIM26/NKRF/SNRPD2 regulatory axis that integrates ubiquitin-mediated proteostasis with transcriptional control of stress granule dynamics. This work provides mechanistic insight into stress-adaptive Osimertinib resistance and identifies potential therapeutic targets for overcoming resistance in EGFR-mutant lung adenocarcinoma.

## Introduction

Lung adenocarcinoma represents the most common histological subtype of non-small cell lung cancer (NSCLC) and remains a leading cause of cancer-related mortality worldwide [1]. Activating mutations in the epidermal growth factor receptor (EGFR) define a clinically important molecular subset of lung adenocarcinoma, for which EGFR tyrosine kinase inhibitors (EGFR-TKIs) have substantially improved patient outcomes [2]. Among these agents, the third-generation EGFR-TKI Osimertinib has become a standard first-line therapy for EGFR-mutant lung adenocarcinoma due to its potent activity against both sensitizing EGFR mutations and the T790M resistance mutation, as well as its favorable toxicity profile [3]. Despite the significant clinical benefits of Osimertinib, most patients eventually develop acquired resistance, leading to disease progression [4].

Acquired resistance to Osimertinib is a multifactorial process that limits the long-term efficacy of EGFR-targeted therapy in EGFR-mutant lung adenocarcinoma [5]. Well-characterized resistance mechanisms include secondary alterations within EGFR itself, such as the C797S mutation, which impairs Osimertinib binding [6]. as well as activation of alternative signaling pathways through receptor tyrosine kinases including MET, AXL and HER2 [7–9]. In addition, phenotypic plasticity and histological transformation have been reported as contributors to therapeutic escape [10, 11]. Increasing evidence suggests that adaptive cellular responses play a critical role in enabling cancer cells to survive sustained targeted therapy. Stress granules, dynamic, non-membranous ribonucleoprotein assemblies that sequester untranslated mRNAs and RNA-binding proteins, have been shown to form in response to targeted therapies and to protect cancer cells by reprogramming protein synthesis and preserving cellular homeostasis [12, 13]. Nevertheless, the role and mechanisms governing stress granule dynamics in the context of Osimertinib resistance remain poorly defined.

Constitutive activation of NF-κB is observed across multiple cancer types and is closely associated with tumor cell survival, proliferation, and therapeutic resistance to therapy [14, 15]. NKRF is a transcriptional repressor initially characterized by its interaction with negative regulatory elements within the promoters of NF-κB target genes [16]. Subsequent studies demonstrated that NKRF can also directly interact with NF-κB family members to suppress NF-κB-dependent transcriptional activity [17, 18]. In addition to its role in inflammatory signaling, NKRF has been described as a stress-responsive regulator of RNA processing during proteotoxic stress [19]. However, whether NKRF governs the expression of stress granule–associated genes and the dynamics of stress granule assembly, and how such regulation influences cancer cell survival under Osimertinib treatment, remains unclear. Moreover, aside from microRNA-mediated post-transcriptional regulation of NKRF [20, 21]. the upstream mechanisms that control NKRF expression and stability in cancer cells under stressed conditions are poorly understood.

## Materials and methods

### Cell culture

The human lung adenocarcinoma cell lines PC9 and HCC827 were obtained from the National Collection of Authenticated Cell Cultures of the Chinese Academy of Sciences in Shanghai, China. PC9OR and HCC827OR cells were kindly provided by Dr Chuan Xu (The First Affiliated Hospital of Nanchang University). Cells were cultured in RPMI-1640 medium supplemented with 10% fetal bovine serum (FBS) and 1% penicillin–streptomycin. Cells were maintained at 37 °C in a humidified atmosphere containing 5% CO₂, and routinely tested to confirm the absence of mycoplasma contamination.

### Plasmids, siRNA, and transfection

Expression plasmids encoding human NKRF, SNRPD2, and TRIM26 were purchased from MiaoLing Plasmid Platform. Site-directed mutagenesis was performed to generate NKRF lysine-to-arginine mutants using as previously described [22]. Empty vector plasmids were used as controls in all overexpression experiments. Small interfering RNAs (siRNAs) targeting NKRF (#SR311038) and TRIM26 (#SR305228), as well as non-targeting scramble siRNAs, were purchased from Origene Technologies. Lentiviral vectors expressing short hairpin RNAs (shRNAs) targeting TRIM26 and NKRF were purchased from Shanghai GeneChem Co., Ltd. and were used to establish stable knockdown cell lines.

Transient transfections of plasmids and siRNAs were performed using MegaTran 2.0 plasmid DNA transfection reagent (#TT210003P5, Origene) and siTran 2.0 siRNA transfection reagent (#TT320002P5, Origene) according to the manufacturers’ instructions. Briefly, cells were seeded and cultured to achieve 60–70% confluence at the time of transfection. Plasmid DNA or siRNA–lipid complexes were prepared in serum-free medium and added to cells. After 24 h, the medium was replaced with complete growth medium. Transfection efficiency was routinely monitored by western blot analysis or quantitative RT–PCR to confirm target gene overexpression or knockdown.

### Cell viability, clonogenic, and migration assays

Cell viability was assessed using Cell Counting Kit-8 reagent (#M4839, AbMole) according to the manufacturer’s instructions. Briefly, cells were seeded into 96-well plates at a density of 3000 cells per well, and treated with Osimertinib or vehicle control (DMSO) for 72 h. At the end of treatment, 10 µL CCK-8 reagent was added to each well and cells were cultured for 1–3 h. The absorbance at 450 nm was measured using a microplate reader (PerkinElmer EnSpire, Shanghai, China). Cell viability was calculated as a percentage relative to vehicle-treated controls, and dose–response curves were generated to determine half-maximal inhibitory concentration (IC₅₀) values.

For clonogenic assays, cells were seeded at a density of 1000 cells per well into 12-well plates and treated with Osimertinib or vehicle control. After 8–10 days of incubation, colonies were fixed with 4% paraformaldehyde and stained with 0.1% crystal violet.

Cell migration was evaluated using wound-healing assays. Cells were seeded into six-well plates and grown to near 100% confluence. A linear wound was generated in the monolayer using a sterile pipette tip, and detached cells were removed by gentle washing with phosphate-buffered saline (PBS). Cells were then cultured in medium containing 1%FBS and Osimertinib or vehicle control. Images of the wound area were captured at 0 h and at indicated time points using an inverted microscope (Invitrogen, EVOS M5000).

### RNA extraction and quantitative real-time PCR (qRT–PCR)

Total RNA was extracted using a column-based RNA Easy Fast Tissue/Cell Kit (#DP451, TIANGEN) according to the manufacturer’s instructions. For cDNA synthesis, equal amounts of total RNA were reverse-transcribed using a reverse transcription kit (#PC7002, Aidlab Biotechnologies). Quantitative real-time PCR was performed using a SYBR Green–based detection kit (#MF013-01, Mei5 Biotechnology) on a real-time PCR instrument (TianLong Gentier96R). Relative gene expression levels were calculated using the 2^^−ΔΔCt^ method, with GAPDH serving as an internal control. The sequences for the primers used in the analysis are provided in Table S1.

### Chromatin immunoprecipitation (ChIP)

Cells were crosslinked with 1% formaldehyde at room temperature to stabilize protein-DNA interactions, and the reaction was quenched with glycine. Cells were then harvested, lysed, and chromatin was sheared to an average fragment size of 200–500 bp by sonication. Sheared chromatin was pre-cleared and incubated overnight at 4 °C with NKRF antibody, or with normal IgG as a negative control. DNA-protein complexes were pulled down using protein A/G magnetic beads (#36417ES08, YEASEN), followed by sequential washes with PBS. Bound chromatin was eluted from the beads, and crosslinks were reversed by heating at 65 °C. DNA was purified and amplified by PCR. Primer sequences used are listed in Table S1. GAPDH promoter region immunoprecipitated by AcH3 antibody was used as positive control.

### Transcriptomic (RNA-seq) analysis

RNA-sequencing analysis was conducted by Wuhan IGeneBook Biotechnology CO., LTD.

### Luciferase reporter assays

The promoter region of human SNRPD2 was amplified by PCR and cloned upstream of the firefly luciferase gene in pGL3 vector. Cells were seeded into 24-well plates and co-transfected with recombinant luciferase reporter plasmid and pRL-TK plasmid. After indicated treatment, cells were lysed, and luciferase activity was measured using Dual-Luciferase® Reporter Assay System (#E1910, Promega Corporation). Firefly luciferase activity was normalized to Renilla luciferase activity, and relative promoter activity was expressed as fold change compared with control groups.

### Immunoprecipitation (IP) and western blot analysis

For western blot analysis, cells were harvested and lysed in ice-cold RIPA lysis buffer (#PR20035, Proteintech) supplemented with protease and phosphatase inhibitors. Protein concentrations were determined using a bicinchoninic acid assay, and equal amounts of total protein were resolved by SDS-PAGE and transferred onto polyvinylidene difluoride (PVDF) membranes. Membranes were blocked with fat-free milk and incubated with primary antibodies against NKRF (#14693-1-AP, Proteintech), GAPDH (#10494-1-AP, Proteintech), Ubiquitin (#10201-2-AP, Proteintech), TRIM26 (#27013-1-AP, Proteintech), SNRPD2 (#14789-1-AP, Proteintech), HA (#51064-2-AP, Proteintech), and Flag (#66008-4-lg, Proteintech) at 4 °C overnight. After incubation with appropriate horseradish peroxidase–conjugated secondary antibodies, immunoreactive bands were visualized using Super ECL Detection Reagent (#36208ES76, YEASEN) and images were captured with a BioRad ChemiDoc MP digital gel image analysis system.

For immunoprecipitation assays, cells were lysed in IP lysis buffer (#G2038, Servicebio), and lysates were incubated with indicated antibodies and protein A/G magnetic beads (#36417ES08, YEASEN) overnight at 4°C with gentle rotation. Immune complexes were then washed with PBS three times. Bound proteins were eluted by boiling in SDS sample buffer and subjected to western blot analysis as described above.

### Immunofluorescence and visualization of stress granules

For immunofluorescence staining, cells were seeded onto glass coverslips and treated as indicated. After treatment, cells were fixed with 4% paraformaldehyde, permeabilized with 0.2% Triton X-100 in PBS, and blocked with 5% bovine serum albumin. Cells were then incubated with primary antibodies against NKRF (#14693-1-AP, Proteintech) and TRIM26 (#sc-393832, Santa Cruz) at 4 °C overnight. Following washing, cells were incubated with fluorophore-conjugated secondary antibodies (#RGAR004, #RGAM002, Proteintech). Nuclei were counterstained with DAPI. Coverslips were mounted using antifade mounting medium prior to imaging. Fluorescence images were acquired using a confocal microscope (Leica Stellaris 5).

For stress granule visualization, cells were transfected with pEF1a-EGFP-TIA1 (#P62639, MiaoLing Plasmid Platform) and pLV2-CMV-G3BP1-mCherry (#P73465, MiaoLing Plasmid Platform) plasmids. After indicated treatment, nuclei were counterstained with Hoechst dye. Stress granule formation and subcellular localization were examined by a confocal microscope (Leica Stellaris 5).

### Cycloheximide (CHX) chase assay

CHX chase assay was performed to determine the half-life of NKRF. Briefly, cells were transfected with the indicated plasmids or siRNAs, and subsequently exposed to 50 µg/mL CHX (#HY-12320, MedChemExpress) to inhibit de novo protein synthesis. Cells were harvested at the indicated time points following CHX addition. To evaluate the contribution of distinct degradation pathways, cells were treated with 10 µM proteasome inhibitor MG-132 (#HY-13259, MedChemExpress) or 10 µM lysosome inhibitor chloroquine (CQ, #HY-17589A, MedChemExpress) in combination with CHX.

### Xenograft models

Male BALB/c nude mice (3–4 weeks old) were purchased from Corues Biotechnology and housed under specific pathogen–free conditions with free access to food and water. All animal experiments were conducted in accordance with institutional guidelines and were approved by the Ethics Committee of the First Affiliated Hospital of Nanchang University (protocol code CDYFY-IACUC-202501GR043). Each mouse was subcutaneously 5 × 10^6^ cells and Osimertinib (15 mg/kg) or vehicle control was intragastrically administered every day. At the experimental endpoint, mice were euthanized, and tumors were excised, measured, and weighed. Tumor volume was calculated using the formula: volume = (length × width²)/2. All experiments were performed in accordance with the approved protocol and guidelines. Investigators were blinded to group allocation during tumor measurement and analysis.

### Immunohistochemistry (IHC) staining

Immunohistochemical staining of xenografted tumors was performed by Wuhan ServiceBio Technology Co., Ltd.

### Statistical analysis

All quantitative data are presented as mean ± standard deviation (SD). Statistical analyses were performed using GraphPad 10.2. Comparisons between two groups were conducted using two-tailed Student’s t tests, while comparisons among multiple groups were performed using one-way or two-way analysis of variance (ANOVA) followed by LSD (Least Significant Difference) test. A *p* value of less than 0.05 was considered statistically significant. All experiments were performed with at least three independent replications, and no data were excluded from the analyses.

## Results

### NKRF is downregulated in Osimertinib-resistant cells and restores sensitivity to Osimertinib in vitro and in vivo

**Fig. 1:**
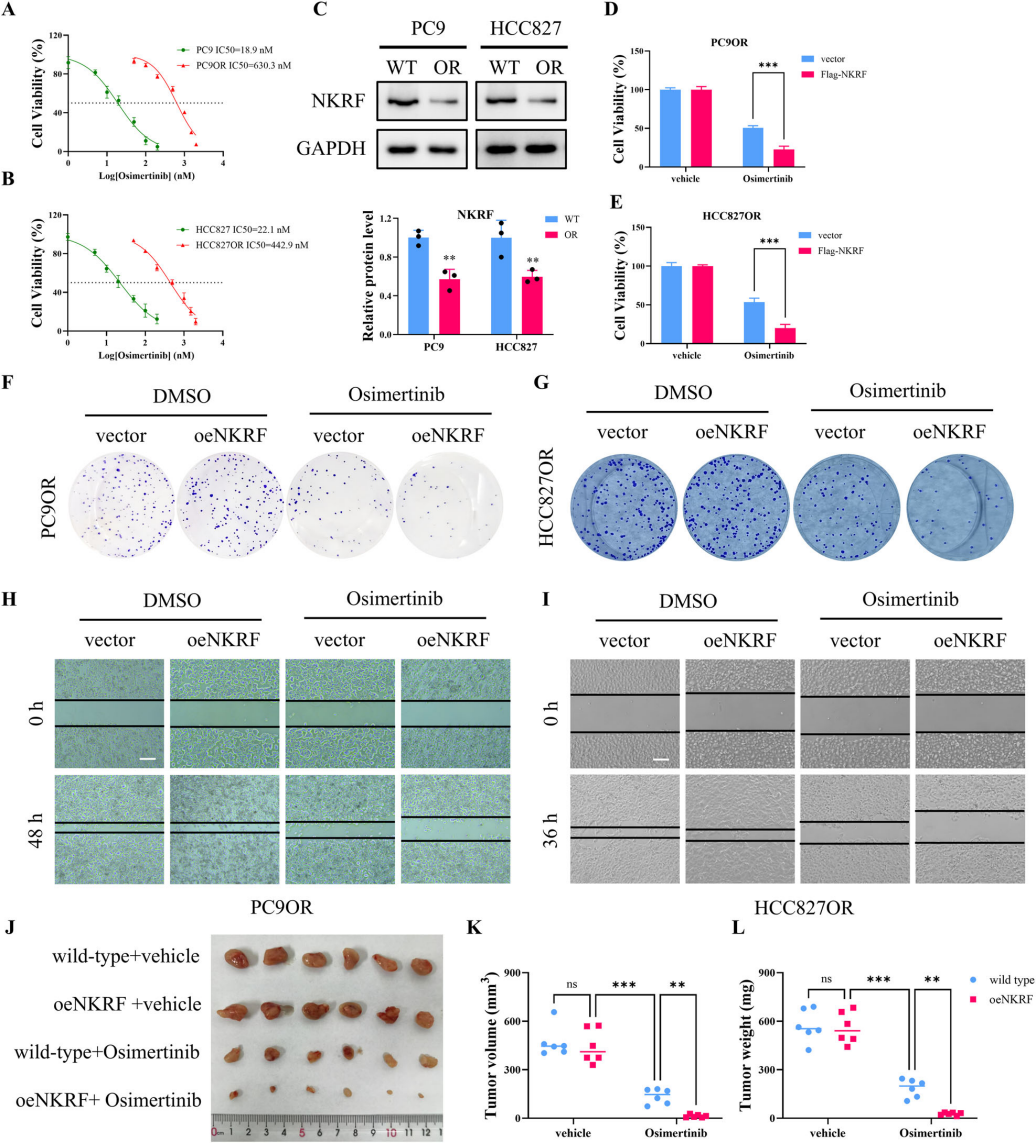
NKRF is downregulated in Osimertinib-resistant cells and restores sensitivity to Osimertinib in vitro and in vivo. **A**, **B** Dose–response curves of parental and Osimertinib-resistant PC9 (PC9OR) and HCC827 (HCC827OR) cells treated with increasing concentrations of Osimertinib. Cell viability was assessed, and IC₅₀ values are shown. **C** Western blot analysis of NKRF protein expression in parental and Osimertinib-resistant PC9 and HCC827 cells. **D**, **E** Cell viability assays of PC9OR and HCC827OR cells transfected with control vector or NKRF-expressing plasmids, followed by treatment with DMSO or Osimertinib. **F**, **G** Representative images and quantification of colony formation assays in PC9OR and HCC827OR cells with or without NKRF overexpression following DMSO or Osimertinib treatment. **H**, **I** Wound-healing assays showing the migratory capacity of PC9OR and HCC827OR cells transfected with control or NKRF plasmids, under DMSO or Osimertinib treatment. Scale bar = 200 µm. **J** Representative images of xenograft tumors derived from PC9OR cells stably expressing control vector or NKRF, treated with vehicle or Osimertinib. **K**, **L** Quantification of tumor volume and weight from the xenograft models. Data are presented as mean ± SD (n = 3). ns *p* > 0.05, **p < 0.01, **p < 0.001.

To establish Osimertinib-resistant models, HCC827 and PC9 cells were chronically exposed to increasing concentrations of Osimertinib, generating resistant derivatives (HCC827OR and PC9OR). Dose–response assays confirmed that both resistant cell lines exhibited markedly increased resistance to Osimertinib compared with their parental counterparts, as reflected by increased IC₅₀ values (Fig. 1A, B). Western blot analysis revealed that NKRF protein expression was substantially decreased in both PC9OR and HCC827OR cells relative to wild-type cells (Fig. 1C). These results indicate an association between NKRF downregulation and acquired resistance to Osimertinib. To determine whether NKRF modulates Osimertinib responsiveness, NKRF was ectopically expressed in resistant cells. Cell viability assays showed that NKRF overexpression had minimal effects on cell viability under vehicle treatment. However, upon Osimertinib exposure, NKRF-overexpressing PC9OR and HCC827OR cells displayed significantly reduced viability compared with vector controls (Fig. 1D, E). Consistently, clonogenic assays demonstrated that NKRF overexpression markedly enhanced the growth-inhibitory effects of Osimertinib in both resistant cell lines, whereas little difference was observed between vector and NKRF-overexpressing cells under DMSO treatment (Fig. 1F, G). Similarly, wound-healing assays revealed that NKRF overexpression significantly impaired cell migration in the presence of Osimertinib, but not under control conditions, in both PC9OR and HCC827OR cells (Fig. 1H, I). The effect of NKRF on Osimertinib sensitivity was further evaluated in vivo using xenograft models. Tumors derived from NKRF-overexpressing cells exhibited comparable growth to controls under vehicle treatment. In contrast, Osimertinib administration resulted in a pronounced reduction in tumor size in the NKRF-overexpressing group compared with wild-type tumors (Fig. 1J). Quantitative analysis confirmed that both tumor volume and tumor weight were significantly decreased in the NKRF-overexpressing group following Osimertinib administration, whereas no significant differences were observed under vehicle conditions (Fig. 1K, L). Collectively, these data demonstrate that NKRF expression is reduced in Osimertinib-resistant lung adenocarcinoma cells and that restoration of NKRF significantly sensitizes resistant cells to Osimertinib both in vitro and in vivo.

### NKRF transcriptionally represses SNRPD2 expression and modulates stress granule formation to regulate Osimertinib resistance

**Fig. 2:**
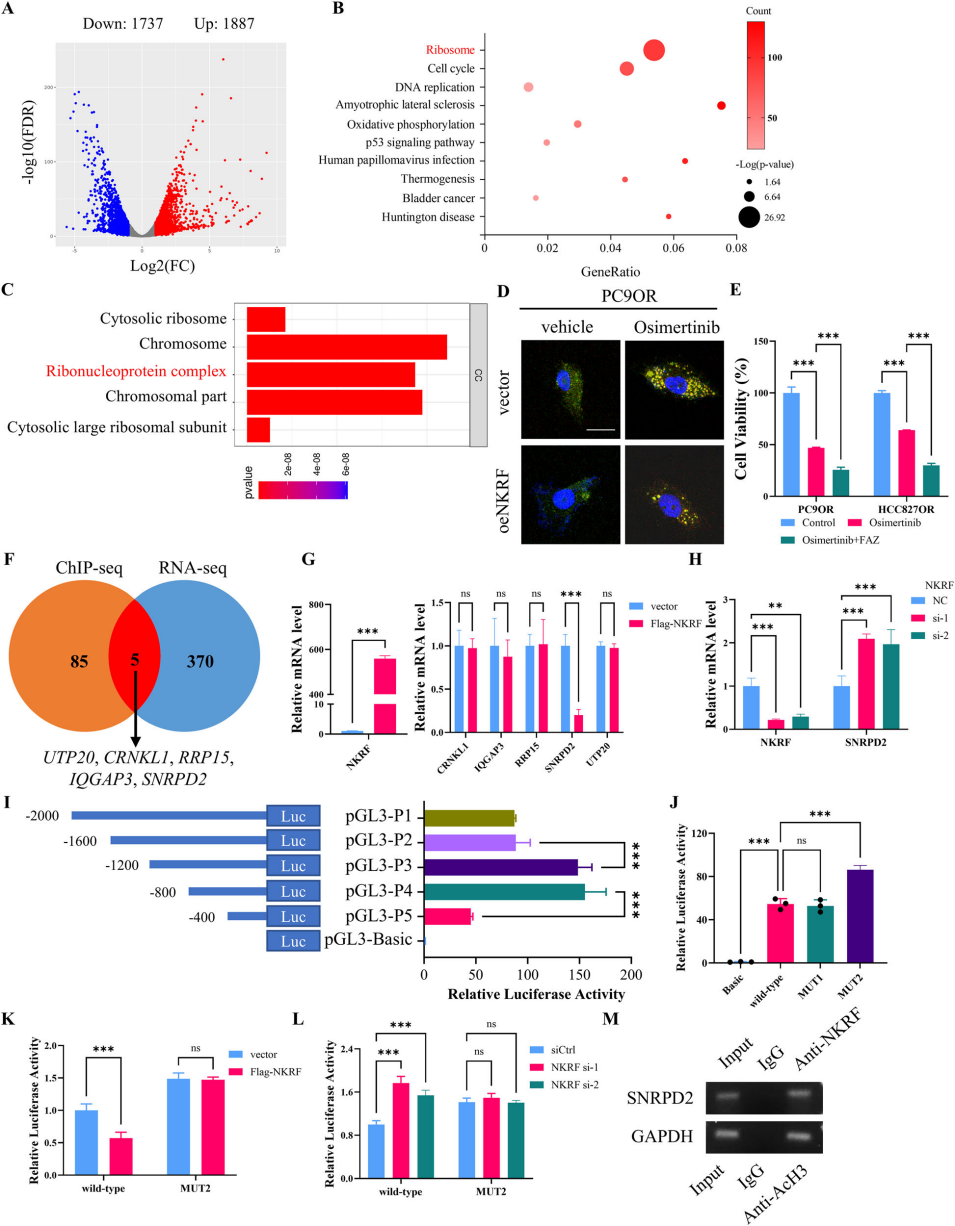
NKRF transcriptionally regulates stress granule–associated genes. **A** Volcano plot of RNA-seq analysis showing differentially expressed genes in PC9OR cells upon NKRF overexpression. Red dots indicate upregulated genes, and blue dots indicate downregulated genes (|log2FC | > 1, FDR < 0.05). **B** KEGG pathway enrichment analysis of differentially expressed genes. Dot size indicates statistical significance, and color intensity represents gene count. **C** GO enrichment analysis of differentially expressed genes. **D** Representative immunofluorescence images showing stress granule formation in PC9OR cells expressing vector or NKRF-expressing plasmid, and treated with vehicle or Osimertinib. Green: EGFP-TIA1, red: G3BP1-mCherry, blue: nuclei. Scale bar = 20 μm. **E** Cell viability assays of PC9OR and HCC827OR cells treated with Osimertinib in the presence or absence of FAZ-3532. **F** Venn diagram showing the overlap between NKRF ChIP-seq targets and NKRF-regulated genes identified by RNA-seq. Five common genes are indicated. **G** qRT–PCR analysis of *NKRF*, *CRNKL1*, *IQGAP3*, *RRP15*, *SNRPD2* and *UTP20* mRNA expression in PC9OR cells transfected with vector or Flag-NKRF plasmid. **H** qRT–PCR analysis of *NKRF* and *SNRPD2* mRNA expression in PC9OR cells transfected with scramble or NKRF-specific siRNAs. **I** Schematic representation and luciferase reporter assays of promoter constructs containing serial truncation of the SNRPD2 promoter region. **J** Luciferase reporter assays of wild-type and mutant SNRPD2 promoter constructs in PC9OR cells. **K** Luciferase reporter assays of wild-type or mutant SNRPD2 promoter reporters in PC9OR cells transfected with vector or Flag-NKRF. **L** Luciferase activity of wild-type or mutant SNRPD2 promoter reporters in cells transfected with control or NKRF siRNAs. **M** ChIP-PCR analysis showing NKRF binding to the SNRPD2 promoter in PC9OR cells. IgG served as a negative control, and GAPDH was used as a positive control. Data are presented as mean ± SD (*n* = 3). ns *p* > 0.05, **p < 0.01, **p < 0.001.

To elucidate the molecular mechanisms by which NKRF regulates Osimertinib resistance, transcriptomic profiling was performed following NKRF modulation. RNA-seq analysis identified 1887 upregulated genes and 1737 downregulated genes upon NKRF overexpression (Fig. 2A). Kyoto Encyclopedia of Genes and Genomes (KEGG) pathway enrichment analysis revealed significant enrichment of ribosome-related pathways, while Gene Ontology (GO) analysis further highlighted ribonucleoprotein complex as prominently affected category (Fig. 2B, C). These findings suggested a potential involvement of stress granule regulation downstream of NKRF. To test our hypothesis, stress granules were visualized by confocal imaging EGFP-TIA1 and mCherry-G3BP1, two canonical stress granule markers. The results demonstrated that NKRF overexpression markedly inhibited the formation of stress granules in PC9OR cells following Osimertinib treatment, compared with vector controls (Fig. 2D). Functionally, pharmacological inhibition of stress granule assembly using 20 µM FAZ-3532 significantly enhanced the growth-inhibitory effect of Osimertinib in both PC9OR and HCC827OR cells (Fig. 2E), supporting a functional role for stress granules in mediating Osimertinib resistance.

To identify direct transcriptional targets of NKRF involved in stress granule regulation, ChIP-seq datasets from ChIP-Atlas were integrated with our RNA-seq data. This analysis identified five overlapping candidate genes: *UTP20*, *CRNKL1*, *RRP15*, *IQGAP3*, and *SNRPD2* (Fig. 2F). Quantitative RT–PCR demonstrated that NKRF overexpression significantly reduced *SNRPD2* mRNA levels, whereas other candidate genes were minimally affected (Fig. 2G). Conversely, siRNA-mediated knockdown of NKRF led to a marked increase in *SNRPD2* mRNA levels (Fig. 2H). To determine whether NKRF directly regulated *SNRPD2* transcription, luciferase reporter assays were performed using a series of SNRPD2 promoter truncations. Deletion of the −1600 to −1200 bp region of the *SNRPD2* promoter led to a significant increase in luciferase activity (Fig. 2I). In silico analysis using the JASPAR database identified two putative NKRF-binding sites within this region, which guided subsequent site-directed mutagenesis of *SNRPD2* promoter (Fig. S1). Mutation of the NKRF-binding Site 2 significantly elevated luciferase activity, whereas mutation of Site 1 had little effect (Fig. 2J). Consistently, NKRF overexpression suppressed wild-type *SNRPD2* promoter activity but failed to repress the MUT2 promoter construct (Fig. 2K). In contrast, NKRF knockdown enhanced wild-type promoter activity without affecting the mutant reporter (Fig. 2L). ChIP assays further confirmed direct occupancy of NKRF at the predicted region within *SNRPD2* promoter (Fig. 2M). These results indicate that NKRF directly suppresses SNRPD2 expression transcription.

**Fig. 3:**
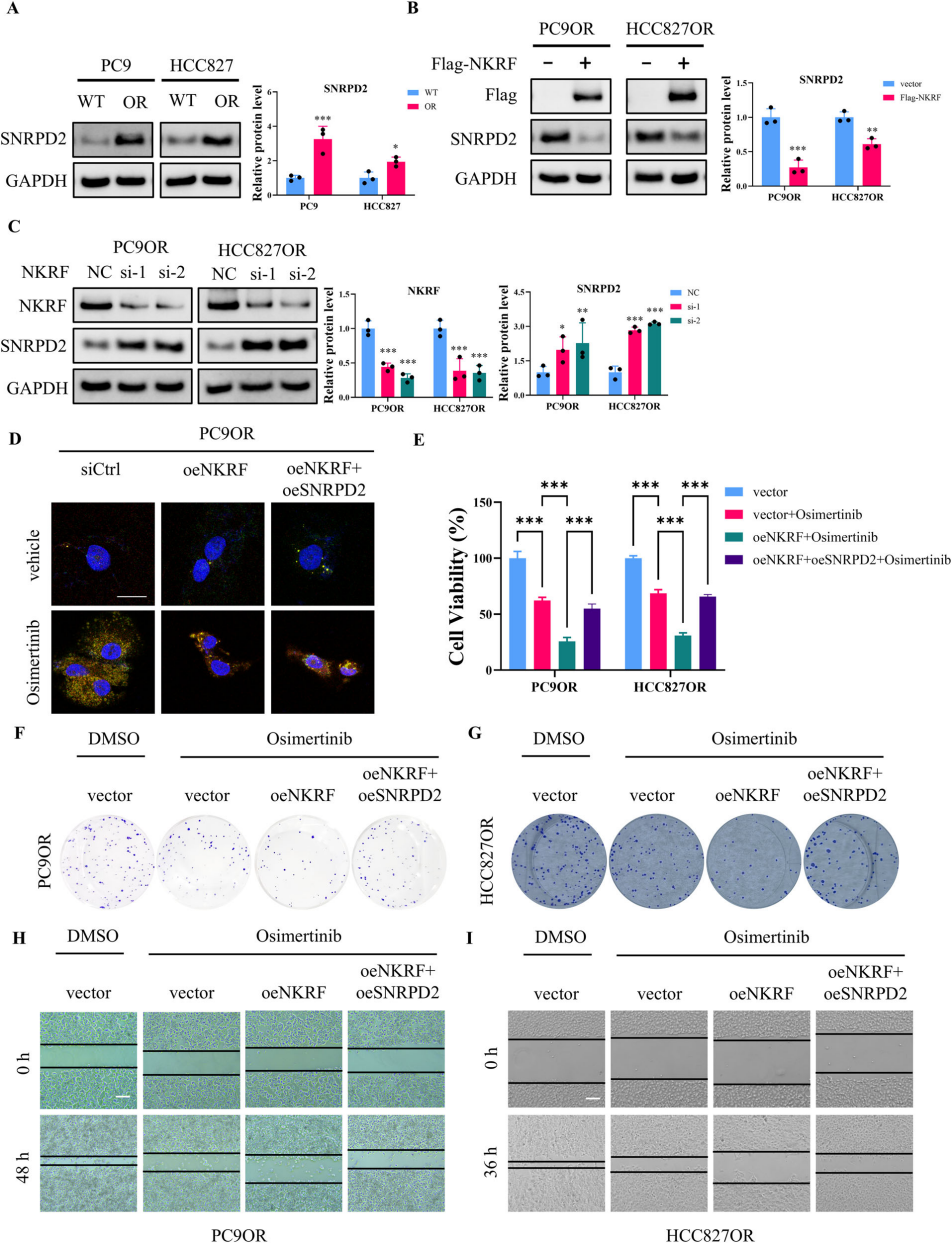
NKRF modulated stress granule formation and Osimertinib resistance via SNRPD2. **A** Western blot analysis of SNPRD2 expression in parental and Osimertinib-resistant PC9 and HCC827 cells. **B** Western blot analysis of SNPRD2 expression in PC9OR and HCC827OR cells transfected with vector or Flag-NKRF plasmid. **C** Western blot analysis of NKRF and SNPRD2 expression in PC9OR and HCC827OR cells transfected with siRNAs targeting NKRF or scramble siRNA (NC). **D** Representative immunofluorescence images showing stress granule formation in PC9OR cells transfected NKRF or SNRPD2 plasmid, and treated with vehicle or Osimertinib. Green: EGFP-TIA1, red: G3BP1-mCherry, blue: nuclei. Scale bar = 20 μm. **E** Cell viability assays of PC9OR and HCC827OR cells transfected with NKRF or SNRPD2 plasmid, and treated with Osimertinib or DMSO. **F**, **G** Clonogenic assays of PC9OR and HCC827OR cells transfected with NKRF or SNRPD2 plasmid, and treated with Osimertinib or DMSO. **H**, **I** Wound healing assays on PC9OR and HCC827OR cells transfected NKRF or SNRPD2 plasmid, and treated with Osimertinib or DMSO. Data are presented as the mean ± SD (*n* = 3). *p < 0.05, **p < 0.01, **p < 0.001.

We further examined the biology function of NKRF-mediated *SNRPD2* transcription. At the protein level, SNRPD2 expression was significantly elevated in PC9OR and HCC827OR cells compared with their parental counterparts (Fig. 3A). NKRF overexpression markedly reduced SNRPD2 protein levels in both resistant cell lines (Fig. 3B), whereas NKRF knockdown resulted in a increase in SNRPD2 expression (Fig. 3C). Functionally, restoration of SNRPD2 expression rescued stress granule formation and cell viability in NKRF-overexpressing PC9OR and HCC827OR cells treated with Osimertinib (Fig. 3D, E). Consistent with these findings, colony formation assays showed that SNRPD2 overexpression attenuated the growth-suppressive effects of NKRF in the presence of Osimertinib (Fig. 3F, G). Similarly, wound-healing assays demonstrated that SNRPD2 restoration partially reversed the inhibitory effects of NKRF on cell migration under Osimertinib treatment in both resistant models (Fig. 3H, I). Collectively, these data identify SNRPD2 as a direct transcriptional target of NKRF and demonstrate that NKRF-mediated repression of SNRPD2 suppresses stress granule formation and Osimertinib resistance.

### TRIM26 interacts with NKRF and promotes its proteasomal degradation through K48-linked ubiquitination

**Fig. 4:**
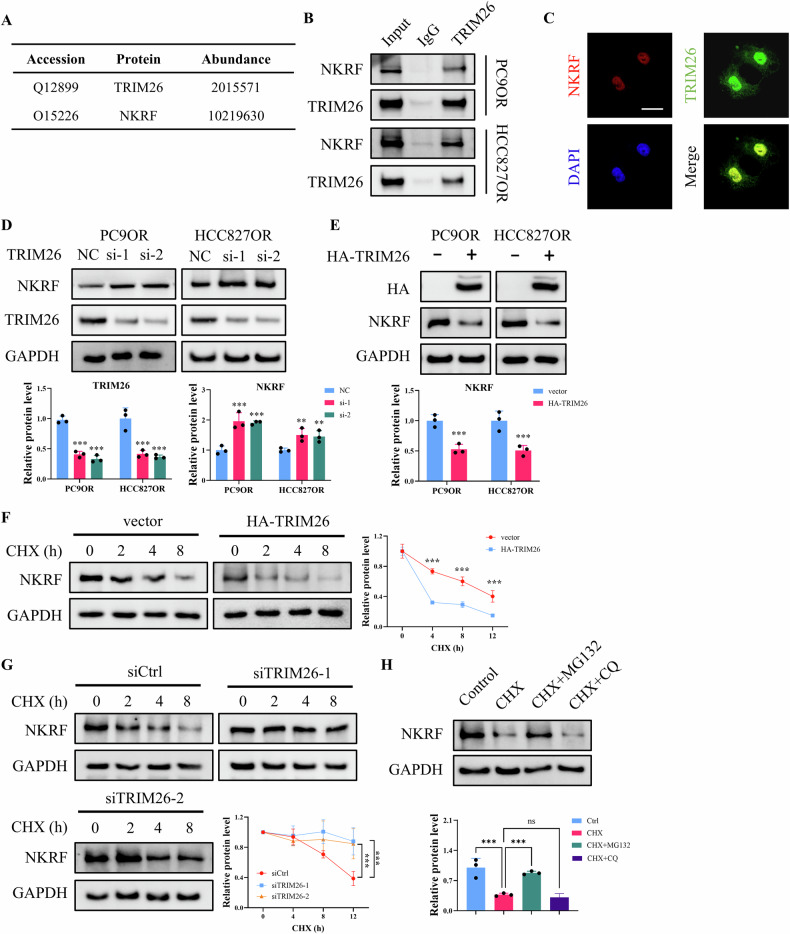
TRIM26 interacts with NKRF and regulates its stability. **A** Accession and abundance of TRIM26 and NKRF identified by IP coupled with mass spectrometry. **B** Co-IP analysis showing the interaction between TRIM26 and NKRF in PC9OR and HCC827OR cells. **C** Fluorescence assay of TRIM26 and NKRF cellular localization in PC9OR cells. Scale bar = 5 μm. **D** Western blot analysis of TRIM26 and NKRF expression in PC9OR and HCC827OR cells transfected with siRNAs targeting TRIM26 or scramble siRNA (NC). **E** Western blot analysis of NKRF expression in PC9OR and HCC827OR cells transfected with vector or HA-TRIM26 plasmid. **F** CHX chase assays of NKRF expression in PC9OR cells transfected with vector or HA-TRIM26. **G** CHX chase assays of NKRF expression in PC9OR cells transfected with siRNA targeting TRIM26 or scramble siRNA (siCtrl). **H** Western blot analysis of NKRF expression in cells treated with CHX, CHX + MG132, or CHX + CQ. Data are presented as the mean ± SD (*n* = 3). **p < 0.01, **p < 0.001.

Using IP coupled with mass spectrometry, our previous study identified NKRF as potential interacting partner of TRIM26 (Fig. 4A). TRIM26 belongs to the TRIM family of E3 ubiquitin ligases and contains a conserved RING domain (16-57 aa) responsible for E3 ligase catalytic activity, followed by B-box motifs (97-138 aa) and a coiled-coil domain (188-227 aa) involved in protein–protein interactions [23]. Specifically, C31 is the key catalytic site of TRIM26 [24]. Western blot analysis indicated that TRIM26 protein expression was substantially elevated in both PC9OR and HCC827OR cells relative to wild-type cells (Fig. S2), suggesting a positive role in Osimertinib resistance. To validate this interaction, co-IP assays were performed and confirmed the association between endogenous NKRF and TRIM26 in both PC9OR and HCC827OR cells (Fig. 4B). Consistently, immunofluorescence analysis revealed nuclear colocalization of NKRF and TRIM26 (Fig. 4C). To investigate whether TRIM26 regulates NKRF expression, TRIM26 was silenced using two independent siRNAs. TRIM26 knockdown led to a marked increase in NKRF protein levels in both PC9OR and HCC827OR cells (Fig. 4D). Conversely, ectopic expression of HA-tagged TRIM26 significantly reduced NKRF protein abundance in both resistant cell lines (Fig. 4E). CHX chase assays further demonstrated that TRIM26 overexpression significantly shortened the half-life of NKRF protein (Fig. 4F), while TRIM26 knockdown markedly stabilized NKRF (Fig. 4G). To clarify the degradation pathway involved, cells were treated with inhibitors of proteasome or lysosome. MG-132, but not CQ, effectively rescued NKRF protein levels following CHX treatment (Fig. 4H), indicating that NKRF degradation occurs primarily through the proteasome-dependent pathway.

**Fig. 5:**
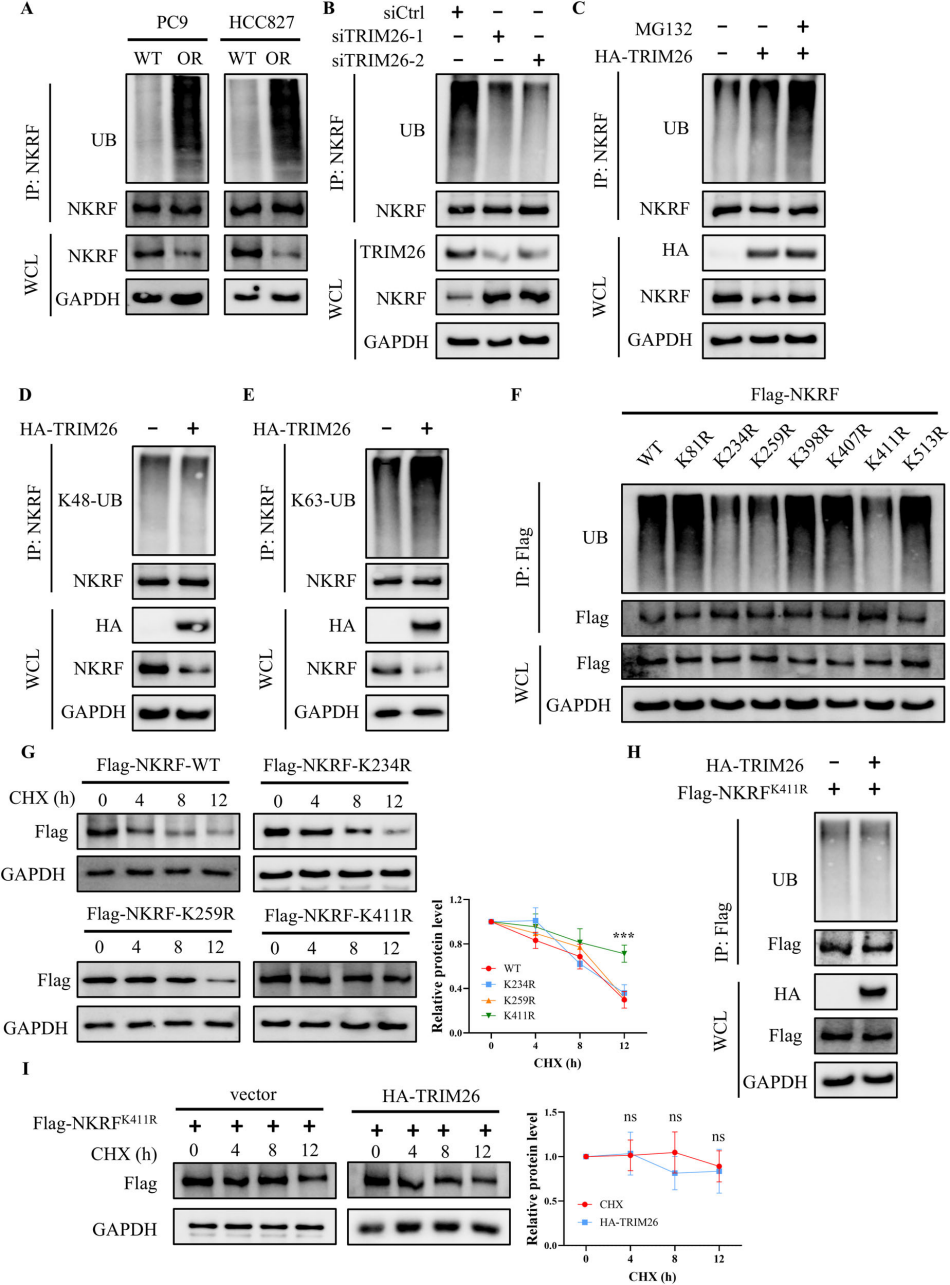
TRIM26 regulates the ubiquitination and stability of NKRF. **A** IP and western blot analysis showing the ubiquitination levels of NKRF in parental and Osimertinib-resistant PC9 and HCC827 cells. **B** IP and western blot analysis of NKRF ubiquitination levels in PC9OR cells transfected with siRNAs targeting TRIM26 or scramble siRNA (siCtrl). **C** IP and western blot analysis of NKRF ubiquitination levels in PC9OR cells transfected with vector or HA-TRIM26 plasmid and treated with MG132. **D**, **E** IP and western blot analysis showing K48-linked and K63-linked polyubiquitin chains attached to NKRF in PC9OR cells transfected with vector or HA-TRIM26 plasmid. **F** IP and western blot analysis showing ubiquitination levels of wild-type and NKRF mutants (K81R, K234R, K259R, K398R, K407R, K411R, K513R) in PC9OR cells. **G** CHX chase assays of wild-type and mutant NKRF expression in PC9OR cells. **H** IP and western blot analysis showing ubiquitination levels of NKRF^K411R^ mutant in PC9OR cells transfected with vector or HA-TRIM26 plasmid. **I** CHX chase assays of NKRF^K411R^ expression in PC9OR cells transfected with vector or HA-TRIM26 plasmid. Data are presented as the mean ± SD (*n* = 3). ns *p* > 0.05, **p < 0.001.

Given that TRIM26 functions as an E3 ubiquitin ligase, we next examined the ubiquitination status of NKRF. Immunoprecipitation assays revealed that NKRF ubiquitination was markedly elevated in Osimertinib-resistant cells compared with parental cells (Fig. 5A). Silencing of TRIM26 significantly reduced NKRF ubiquitination (Fig. 5B), whereas TRIM26 overexpression enhanced NKRF ubiquitination, particularly upon proteasome inhibition (Fig. 5C). Importantly, overexpression of neither TRIM26-ΔRING nor TRIM26-C31S mutant enhanced NKRF ubiquitination (Fig. S3), indicating TRIM26 promotes NKRF ubiquitination in a RING domain–dependent manner. To determine the type of ubiquitin linkage involved, K48- and K63-specific polyubiquitin antibodies were employed. These analyses demonstrated that TRIM26 predominantly promoted K48-linked, but not K63-linked, polyubiquitination of NKRF (Fig. 5D, E). The candidate ubiquitination sites of NKRF were selected based on ubiquitination annotations from PhosphoSitePlus. Only lysine residues supported by more than one independent reference were included for site-directed mutagenesis. Ubiquitination analysis identified Lys234, Lys259 and Lys411 as potential ubiquitination sites, as mutation of these residues markedly reduced overall NKRF ubiquitination (Fig. 5F). Notably, CHX chase assays demonstrated that NKRF^K411R^ exhibited significantly enhanced stability compared with wild-type NKRF and the NKRF^K234R^, NKRF^K259R^ mutants (Fig. 5G). Further analysis showed that TRIM26-mediated ubiquitination of NKRF was largely abolished in the K411R mutant (Fig. 5H), and TRIM26 overexpression failed to accelerate degradation of NKRF^K411R^ mutant (Fig. 5I). Together, these results demonstrate that TRIM26 directly interacts with NKRF and promotes its proteasomal degradation via K48-linked ubiquitination at Lys411.

### TRIM26 promotes Osimertinib resistance through the NKRF/SNRPD2 axis in vitro and in vivo

**Fig. 6:**
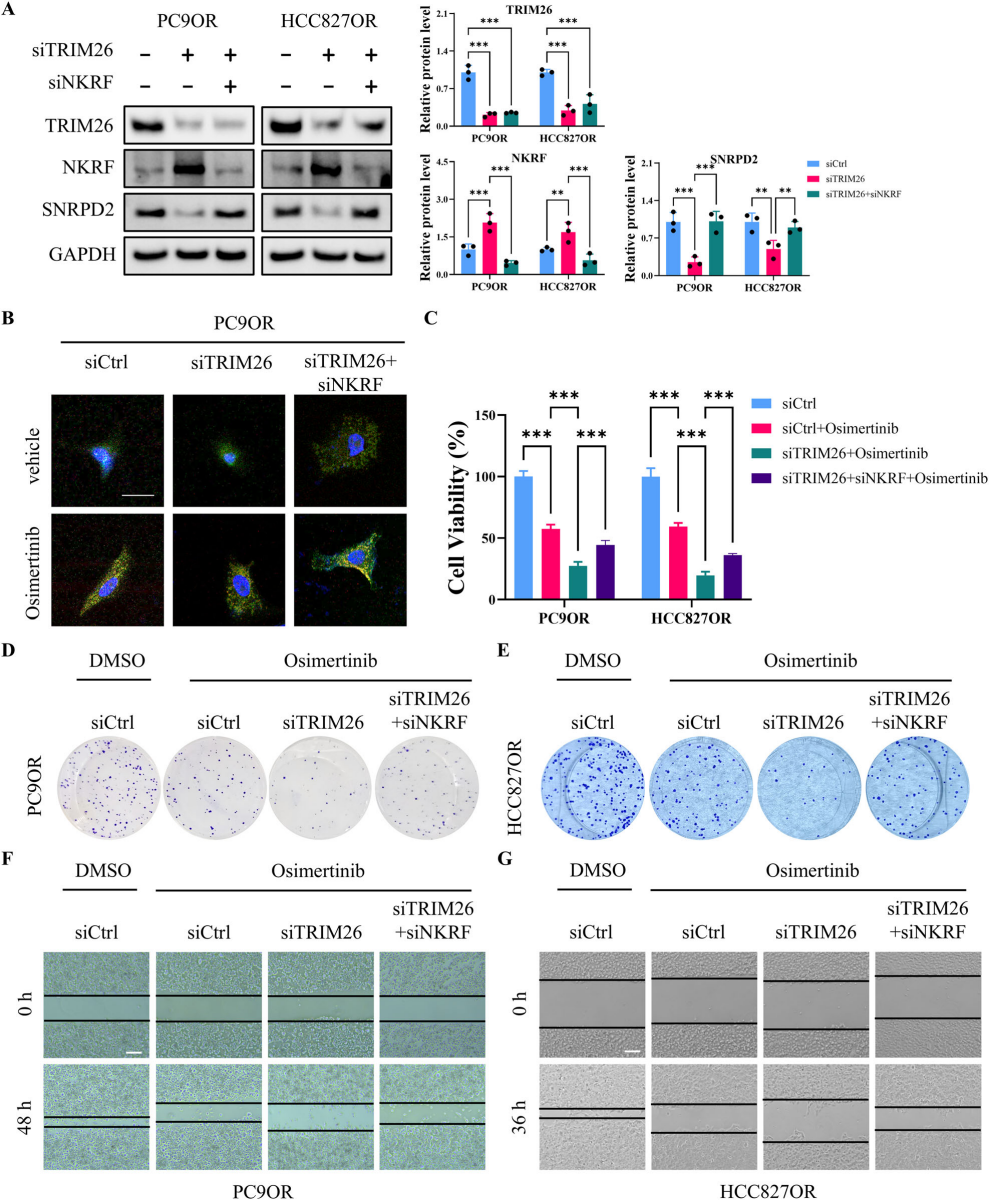
TRIM26 knockdown enhances Osimertinib sensitivity through the modulation of NKRF expression. **A** Western blot analysis showing the expression of TRIM26, NKRF, and SNRPD2 in PC9OR and HCC827OR cells transfected with siRNA targeting TRIM26 and NKRF. **B** Representative immunofluorescence images showing stress granule formation in PC9OR cells transfected siRNA targeting TRIM26 and NKRF, and treated with vehicle or Osimertinib. Green: EGFP-TIA1, red: G3BP1-mCherry, blue: nuclei. Scale bar = 20 μm. **C** Cell viability assays of PC9OR and HCC827OR cells transfected with siRNA targeting TRIM26 or NKRF, and treated with Osimertinib or DMSO. **D**, **E** Clonogenic assays of PC9OR and HCC827OR cells transfected with siRNA targeting TRIM26 or NKRF, and treated with DMSO or Osimertinib. **F**, **G** Wound healing assays of PC9OR and HCC827OR cells transfected with siRNA targeting TRIM26 or NKRF, and treated with DMSO or Osimertinib. Data are presented as the mean ± SD (*n* = 3). **p < 0.01, **p < 0.001.

To determine whether TRIM26 regulates Osimertinib resistance through NKRF and its downstream effector SNRPD2, epistasis analyses were performed in PC9OR and HCC827OR cells. Silencing of TRIM26 markedly increased NKRF protein levels while concomitantly reducing SNRPD2 expression. Notably, simultaneous knockdown of NKRF largely reversed the TRIM26 depletion–induced suppression of SNRPD2 in both resistant cell lines (Fig. 6A), indicating that TRIM26 regulates SNRPD2 expression in an NKRF-dependent manner. Consistently, confocal imaging revealed that TRIM26 knockdown impaired stress granule formation in PC9OR cells under Osimertinib treatment, whereas co-silencing NKRF restored stress granule assembly (Fig. 6B). Functionally, TRIM26 depletion significantly enhanced cellular sensitivity to Osimertinib in both PC9OR and HCC827OR cells. This sensitizing effect was partially abrogated by simultaneous NKRF knockdown (Fig. 6C), supporting a functional requirement for NKRF in TRIM26-mediated drug resistance. Consistent with these observations, colony formation assays showed that TRIM26 silencing markedly potentiated the growth-inhibitory effects of Osimertinib, while co-silencing NKRF restored clonogenic survival in both resistant models (Fig. 6D, E). Similarly, wound-healing assays revealed that TRIM26 knockdown significantly impaired cell migration in the presence of Osimertinib, whereas NKRF depletion attenuated this inhibitory effect (Fig. 6F, G).

**Fig. 7:**
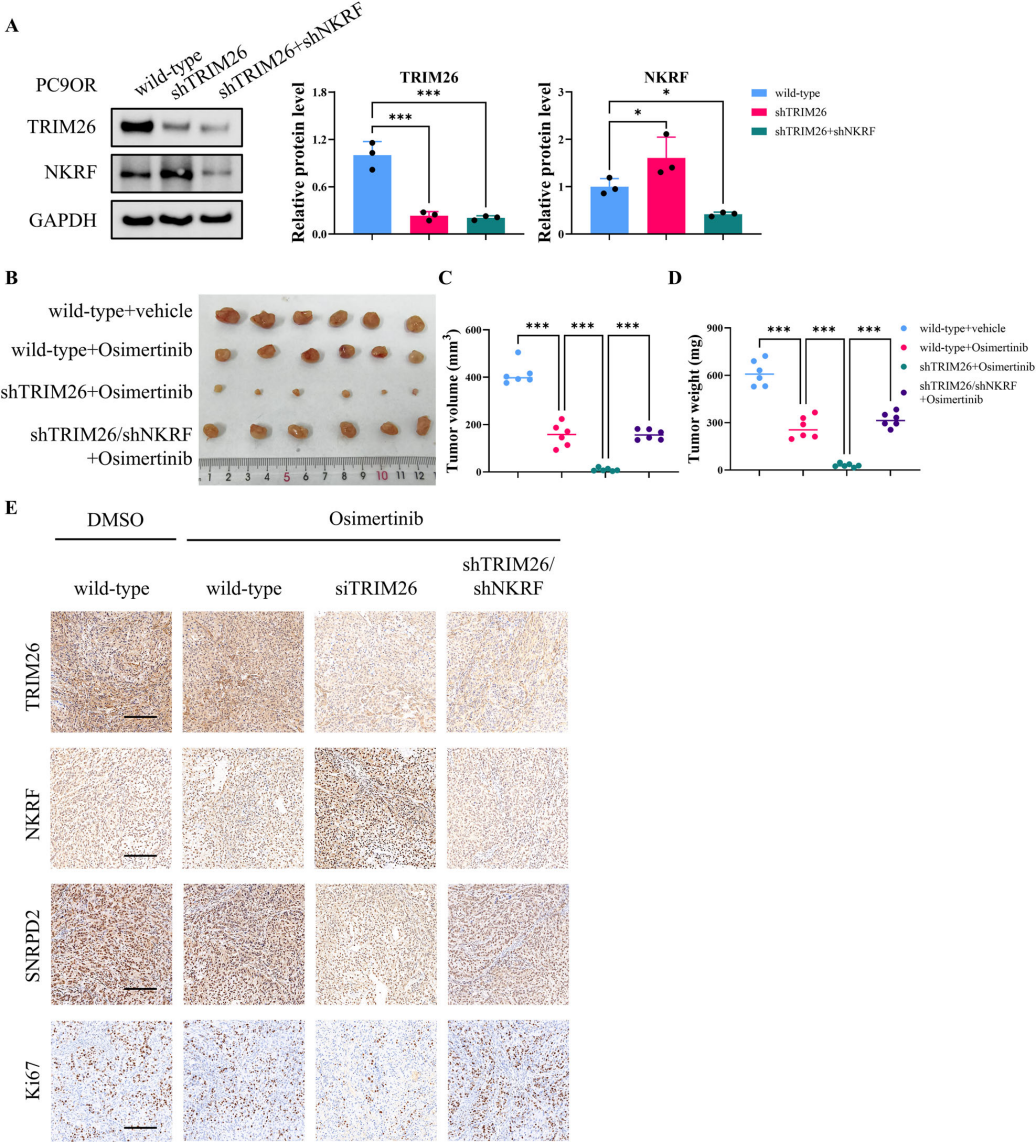
TRIM26 silencing reduces Osimertinib resistance through NKRF modulation in vivo. **A** Western blot analysis showing the expression of TRIM26 and NKRF in wild-type, shTRIM26, and shTRIM26+shNKRF-transduced PC9OR cells. **B** Representative images of tumors from wild-type, shTRIM26, and shTRIM26+shNKRF-transduced PC9OR xenografts treated with vehicle or Osimertinib. **C**, **D** Quantification of tumor volume and weight from the xenograft models. **E** IHC staining of the xenografted tumor sections in each group. Scale bar = 200 μm. Data are presented as the mean ± SD (*n* = 6). *p < 0.05, **p < 0.001.

The in vivo relevance of the TRIM26-NKRF axis was further examined using xenograft models established with PC9OR cells stably expressing shRNAs targeting TRIM26, with or without concurrent NKRF knockdown. Efficient depletion of TRIM26 and NKRF in the stable cell lines was confirmed by western blot analysis (Fig. 7A). Under Osimertinib administration, tumors derived from shTRIM26 cells exhibited a pronounced reduction in size compared with control tumors. This effect was significantly reversed upon simultaneous NKRF knockdown (Fig. 7B). Quantitative analyses confirmed that both tumor volume and tumor were significantly decreased in the shTRIM26 group following Osimertinib treatment, whereas co-silencing NKRF partially restored tumor growth (Fig. 7C, D). Immunohistochemical analysis of xenograft tissues revealed decreased expression of SNRPD2 and the proliferation marker Ki67 in the shTRIM26 group, both of which were restored in tumors derived from shTRIM26+shNKRF cells (Fig. 7E). Collectively, these data demonstrate that TRIM26 promotes Osimertinib resistance by suppressing NKRF, thereby sustaining SNRPD2 expression. Disruption of TRIM26 restores NKRF expression and sensitizes resistant lung adenocarcinoma cells to Osimertinib both in vitro and in vivo.

## Discussion

In this study, we identify NKRF as a previously unrecognized regulator of Osimertinib resistance in lung adenocarcinoma and elucidate a mechanistic axis linking NKRF stability, stress granule regulation, and therapeutic response to Osimertinib. NKRF has traditionally been studied in the context of inflammatory signaling [25, 26]. Its role in tumor biology remains limited and context dependent. Prior studies have reported tumor-suppressive functions of NKRF in triple-negative breast cancer and gastric cancer [21, 27]. whereas a pro-survival role conferring cisplatin resistance has been described in colorectal cancer [28]. Our findings extend the functional repertoire of NKRF to include regulation of Osimertinib resistance. The consistent downregulation of NKRF observed in two Osimertinib-resistant cell line, together with the pronounced re-sensitization achieved by NKRF restoration, indicates that loss of NKRF is a critical driver of acquired resistance. Notably, modulation of NKRF expression exerted minimal effects on basal cell viability in the absence of Osimertinib treatment, yet profoundly influenced cellular responses under Osimertinib exposure. This context-dependent phenotype suggests that NKRF primarily constrains stress-adaptive survival programs engaged during targeted therapy rather than functioning as a general growth regulator. Such a role is consistent with emerging models of drug resistance, in which cancer cells exploit stress-response pathways to survive therapeutic pressure [29].

Stress granules are dynamic, non-membranous ribonucleoprotein complexes that form in response to diverse cellular stresses [30]. Accumulating evidence indicates that stress granules play critical roles in promoting tumor cell proliferation, migration, and metastasis [31–33]. Over the past years, a close association between stress granule formation and chemoresistance has been increasingly recognized across multiple cancer types, including doxorubicin resistance in triple-negative breast cancer, docetaxel in prostate cancer, and capecitabine resistance in gastric cancer [34–36]. Consistent with these observations, we found that stress granule formation was remarkedly increased following Osimertinib treatment in resistant cells. Pharmacological inhibition of stress granule assembly re-sensitized resistant cells to Osimertinib, highlighting the critical role for stress granules in mediating resistance to Osimertinib. Mechanistically, stress granule assembly is initiated by phosphorylation of eIF2α, leading to translational arrest and accumulation of untranslated mRNAs. These stalled mRNAs recruit RNA-binding proteins, such as G3BP1/2, TIA1, and CAPRIN1, which nucleate stress granule assembly via multivalent RNA-protein and protein-protein interactions [37, 38]. SNRPD2, a core component of ribonucleoprotein complexes, has been implicated in RNA processing and ribonucleoprotein organization [39]. Notably, elevated SNRPD2 expression has been reported in multiple cancer types and is associated with malignant phenotypes [40, 41]. However, its role in stress granule dynamics and therapeutic resistance has remained largely unexplored. In this study, we identify SNRPD2 as a key effector linking stress granule formation to Osimertinib resistance. We demonstrate that SNRPD2 expression is upregulated in Osimertinib-resistant cells, and that NKRF directly represses SNRPD2 transcription, thereby restraining stress granule assembly. Importantly, ectopic expression of SNRPD2 restored stress granule formation and conferred Osimertinib resistance in NKRF-overexpressing cells. Recent work has highlighted SNRPD2 as a potential therapeutic target in cancer, demonstrating that perturbation of SNRPD2 impairs tumor cell viability and sensitizes cancer cells to stress [42]. These findings are consistent with our observation that elevated SNRPD2 promotes stress granule assembly and Osimertinib tolerance, further supporting the translational relevance of targeting SNRPD2 in EGFR-mutant lung adenocarcinoma.

TRIM26 is a member of tripartite motif protein family, characterized by a conserved N-terminal RING domain that confers E3 ligase activity, as well as B-box motifs and a coiled-coil region that mediates protein oligomerization [43]. Initially identified as a regulator of innate immune signaling through ubiquitin-dependent degradation of IRF3 [24, 44]. TRIM26 has since merged as a context-dependent modulator of tumor biology, exerting divergent functions depending on its cellular context and substrate specificity. In several malignancies, including renal cell carcinoma, osteosarcoma, and hepatocellular carcinoma, TRIM26 has been reported to function as a tumor suppressor [45–47]. In contrast, oncogenic roles for TRIM26 have been described in non-small cell lung cancer, bladder cancer, and glioma [48–50]. In the present study, we identify TRIM26 as a direct interacting partner of NKRF and demonstrate that TRIM26 promotes NKRF degradation through the ubiquitin–proteasome system. Mechanistically, TRIM26 facilitates K48-linked polyubiquitination of NKRF, with Lys411 identified as a critical ubiquitination site required for TRIM26-mediated NKRF turnover. This site-specific regulation underscores the precision of TRIM26-NKRF interaction and provides a mechanistic explanation for the reduced NKRF protein levels observed in Osimertinib-resistant cells. Recent studies have further implicated TRIM26 in therapeutic responses across multiple cancer types. TRIM26 depletion has been shown to sensitize gastric cancer cells to cisplatin by enhancing oxidative stress and ferroptosis [51]. whereas TRIM26 knockdown was associated with enhanced responsiveness to doxorubicin treatment in triple-negative breast cancer [52]. Consistent with these findings, our data demonstrate that disruption of TRIM26 impairs stress granule formation and markedly enhances sensitivity to Osimertinib both in vitro and in vivo. Importantly, epistasis analyses reveal that the effects of TRIM26 depletion on Osimertinib response are largely dependent on NKRF and its downstream effector SNRPD2. Integrated genetic and epigenetic regulation has recently been shown to shape susceptibility and progression of lung adenocarcinoma [53]. Together with our findings, these data delineate a linear TRIM26-NKRF-SNRPD2 regulatory axis that integrates ubiquitin-mediated proteostasis with transcriptional control of stress adaptation, thereby contributing to acquired resistance to EGFR-targeted therapy.

In conclusion, this study identifies TRIM26 as an upstream E3 ligase that destabilizes NKRF and delineates the downstream consequences of NKRF loss on SNRPD2 expression and stress granule formation under Osimertinib treatment. These results highlight the critical contribution of transcriptional and post-transcriptional stress-response pathways to therapeutic resistance and suggest that targeting the TRIM26-NKRF-SNRPD2 axis may provide a rational strategy to restore Osimertinib sensitivity and improve the durability of targeted therapy in EGFR-mutant lung adenocarcinoma.

## Supplementary information

Supplemental Figures

Supplemental Table

Original Western Blots Data

## Data Availability

The data that support the findings of this study are available from the corresponding author upon reasonable request.
