# Supplementary material for: TRIM26-mediated NKRF degradation drives Osimertinib resistance through SNRPD2-dependent stress granule formation in lung adenocarcinoma
Source: Cell Death Dis. 2026 Apr 24;17(1):541. doi: 10.1038/s41419-026-08787-x (PMC13237084; doi:10.1038/s41419-026-08787-x)
Supplement: Supplementary file 1 — Supplemental Figures [file 41419_2026_8787_MOESM1_ESM.pdf]

Supplemental Figure 1

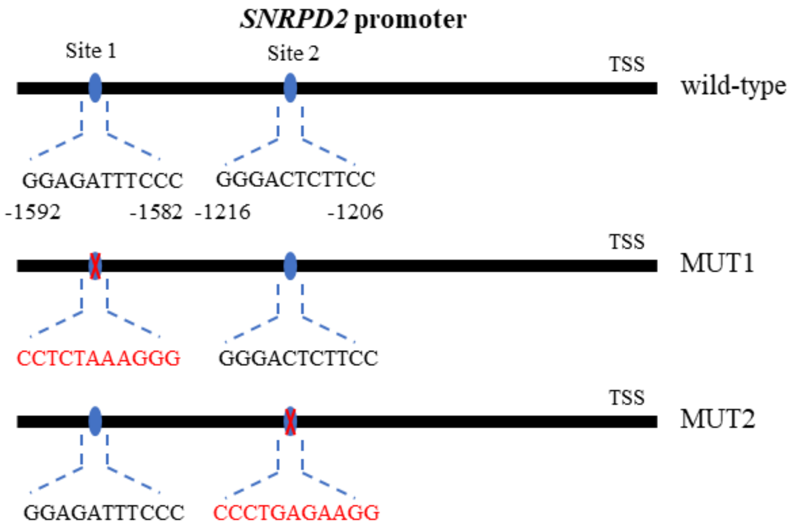

Supplemental Figure 1. Schematic representation of NKRF binding sites on *SNRPD2* promoter and the construction of mutant reporter.

Supplemental Figure 2

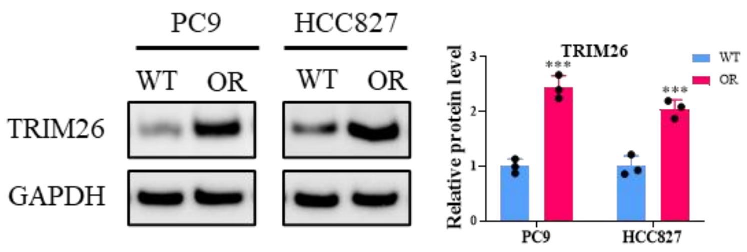

Supplemental Figure 2. Western blot analysis of TRIM26 protein expression in parental and Osimertinib-resistant PC9 and HCC827 cells. Data are presented as mean ± SD (n=3). ***$p < 0.001$.

Supplemental Figure 3

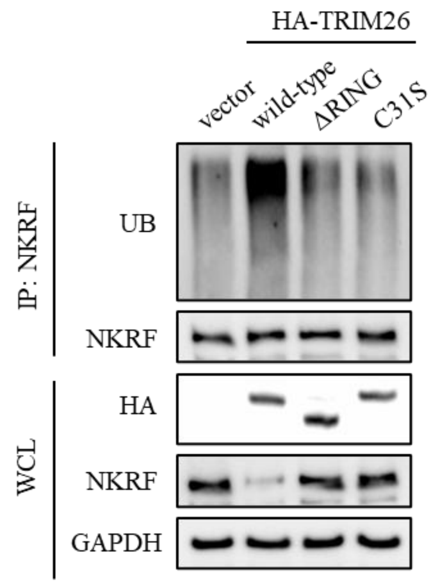

Supplemental Figure 3. TRIM26 promotes NKRF ubiquitination in a RING domain-dependent manner. IP and western blot analysis of NKRF ubiquitination levels in PC9OR cells transfected with vector, wild-type TRIM26, RING domain-deleted TRIM26 (ΔRING), or E3 ligase-deficient TRIM26 (C31S) plasmid.
