# Supplementary material for: TRIM26-mediated NKRF degradation drives Osimertinib resistance through SNRPD2-dependent stress granule formation in lung adenocarcinoma
Source: Cell Death Dis. 2026 Apr 24;17(1):541. doi: 10.1038/s41419-026-08787-x (PMC13237084; doi:10.1038/s41419-026-08787-x)
Supplement: Supplementary file 2 — Supplemental Table [file 41419_2026_8787_MOESM2_ESM.pdf]

Supplemental Table. Primers used in this study.

Quantitative RT-PCR	
TRIM26-F	5’-AGTTGTGCGAGCGACACCGAGA-3’
TRIM26-R	5’-TCCCTTTGCCTGGAAGCCCTGA-3’
CRNKL1-F	5’- AGCACGGCGTTACCCACCTCAT -3’
CRNKL1-R	5’- CGGAAGCGGAACTTGCAGGACT -3’
IQGAP3-F	5’- GCTGTGCTGTGGCTTGAGGAGA-3’
IQGAP3-R	5’- GCTGTGTCTGCTGGACGCTGTT-3’
RRP15-F	5’- TGGTAACTGGAGCCGTAGCGTC-3’
RRP15-R	5’- CCATAGCATCTGCCCAGCCCAT-3’
SNRPD2-F	5’- TGCAGAAGCGAGAGGAGGAGGA-3’
SNRPD2-R	5’- TCCGCAGGACCACGATGACTGA-3’
UTP20-F	5’- GCTTCGGCTGCTGCATCTGAGT-3’
UTP20-R	5’- AGCGGGAACCTCAAGACCCAGA-3’
GAPDH-F	5’- TCCATGCCATCACTGCCACCCA-3’
GAPDH-R	5’- GACGCCTGCTTCACCACCTTCT-3’
ChIP-PCR	
SNRPD2-F	5’-AAGGCTCAGGAAAAGTCCTTCC-3’
SNRPD2-R	5’-TAAGGCTTCGGGCTGGTTCTGC-3’
GAPDH-F	5’- CAGGCTGGATGGAATGAAAGG-3’
GAPDH-R	5’- GCACGGAAGGTCACGATGT-3’
