# Supplementary material for: TRIM26-mediated NKRF degradation drives Osimertinib resistance through SNRPD2-dependent stress granule formation in lung adenocarcinoma
Source: Cell Death Dis. 2026 Apr 24;17(1):541. doi: 10.1038/s41419-026-08787-x (PMC13237084; doi:10.1038/s41419-026-08787-x)

Figure 1

A

NKRF GAPDH NKRF GAPDH

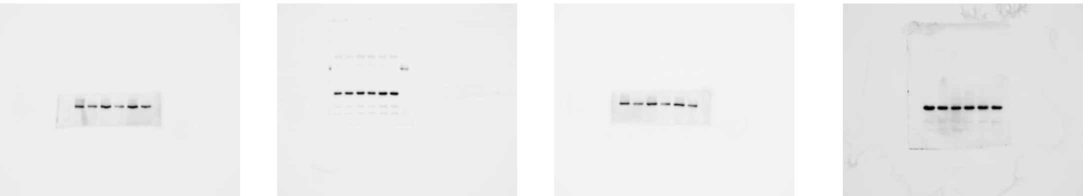

Figure 3

B

SNRPD2 GAPDH SNRPD2 GAPDH

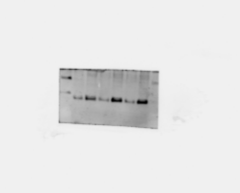
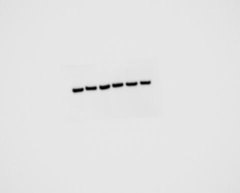
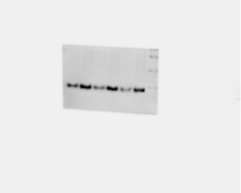

C

SNRPD2 NKRF GAPDH

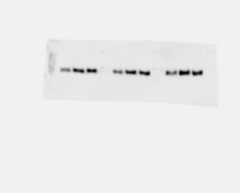
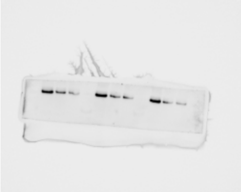
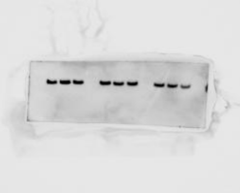

SNRPD2 NKRF GAPDH

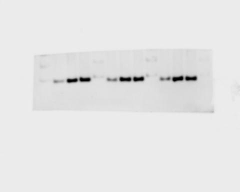
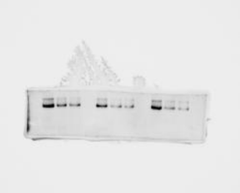
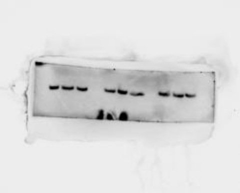

Figure 4

B

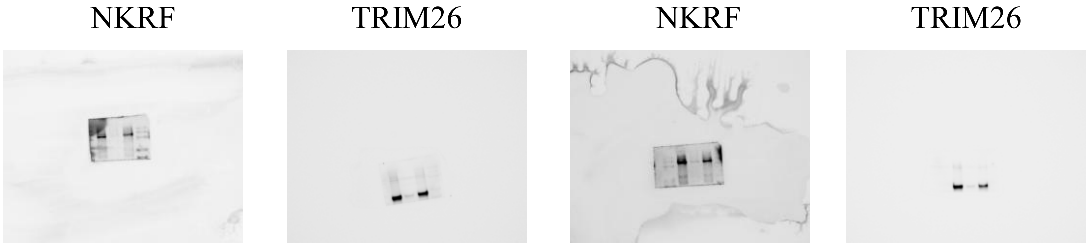

D

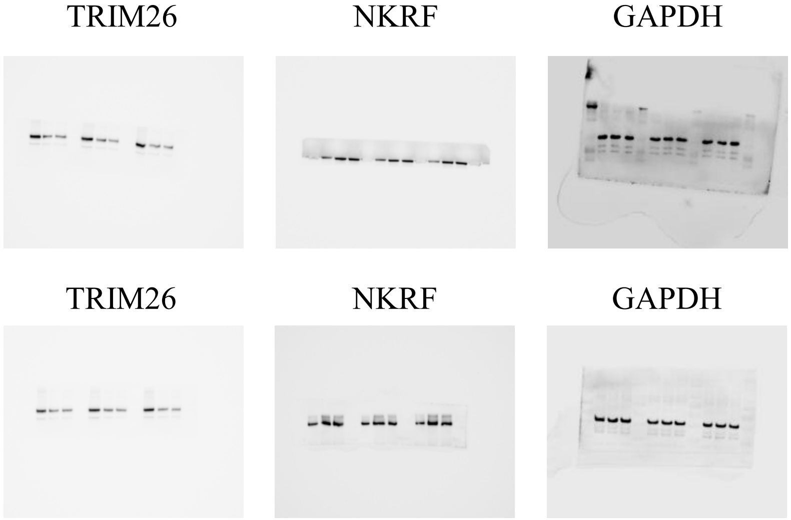

E

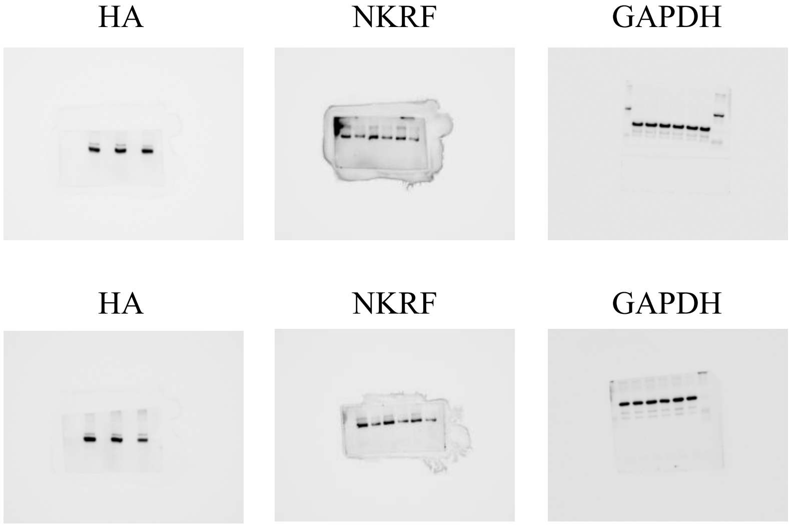

F

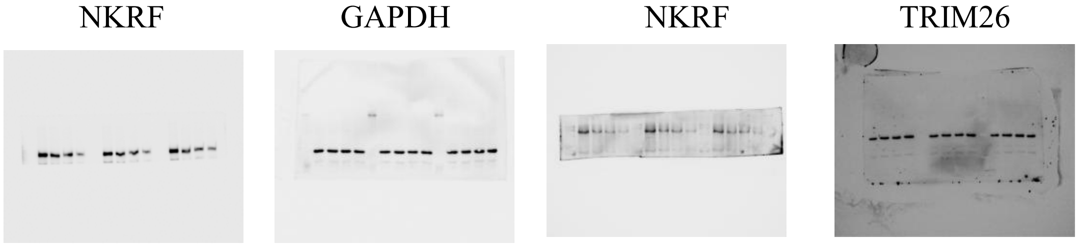

Figure 4

G

NKRF NKRF NKRF

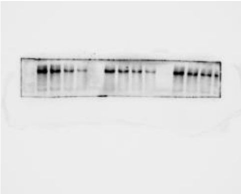 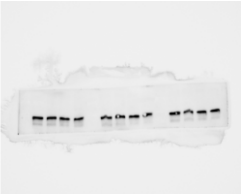 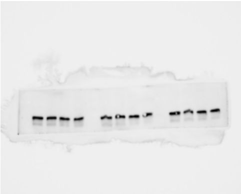

GAPDH GAPDH GAPDH

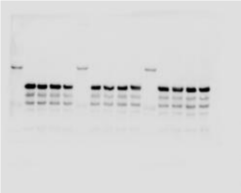 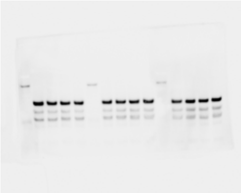 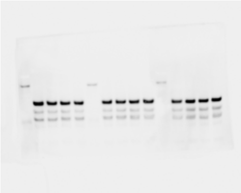

H

NKRF GAPDH

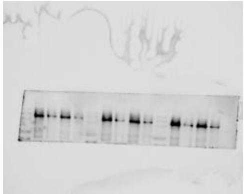 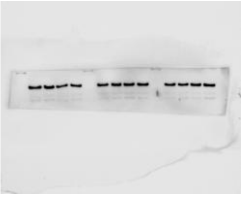

Figure 5

A

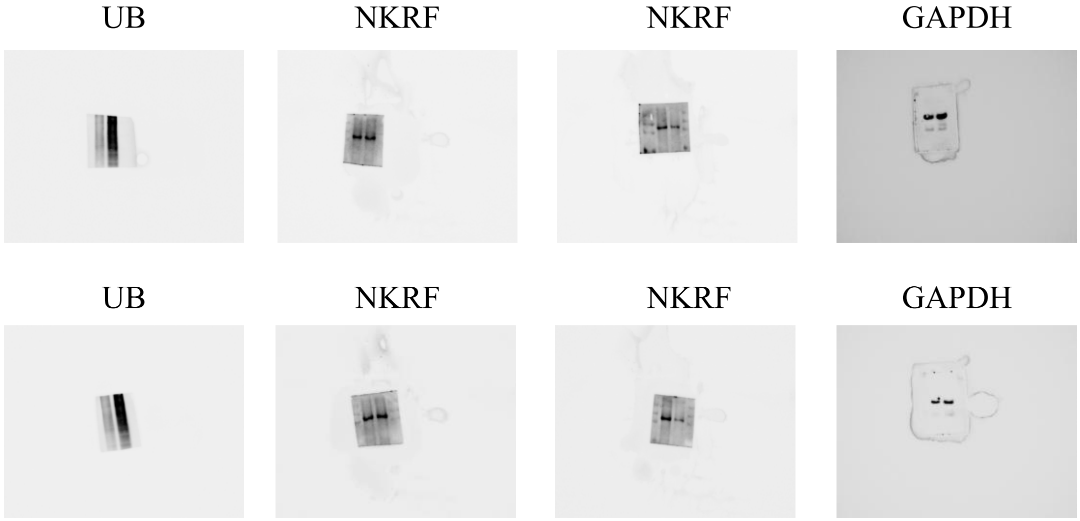

B

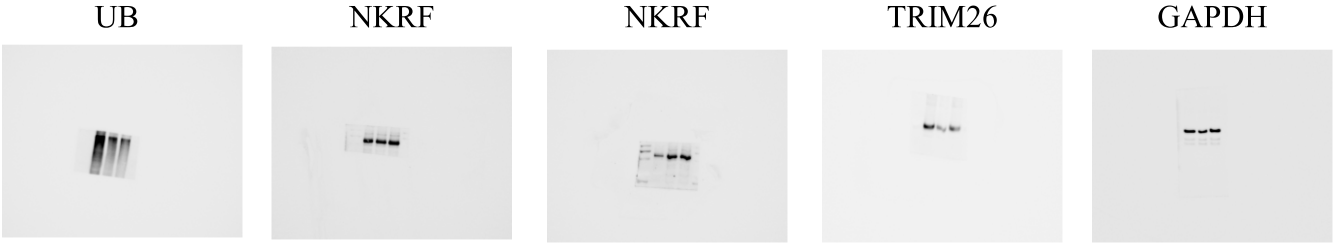

C

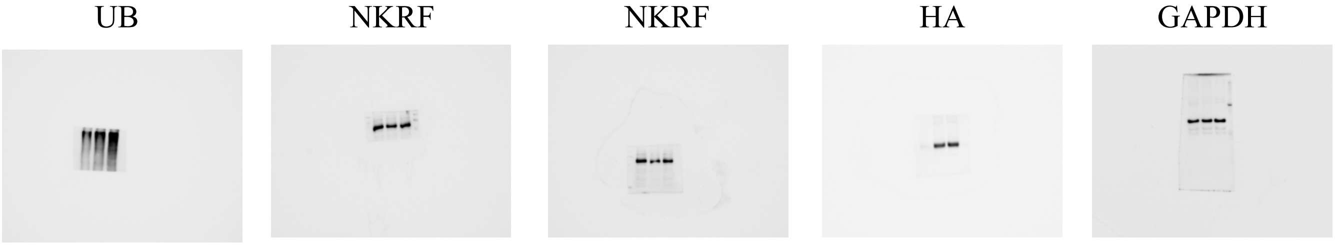

D

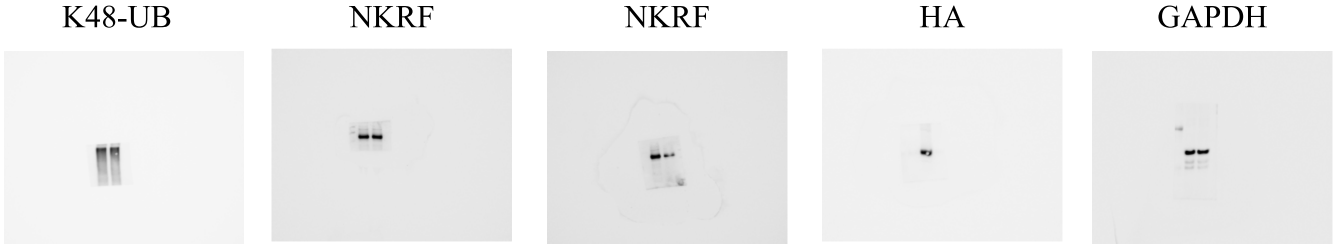

E

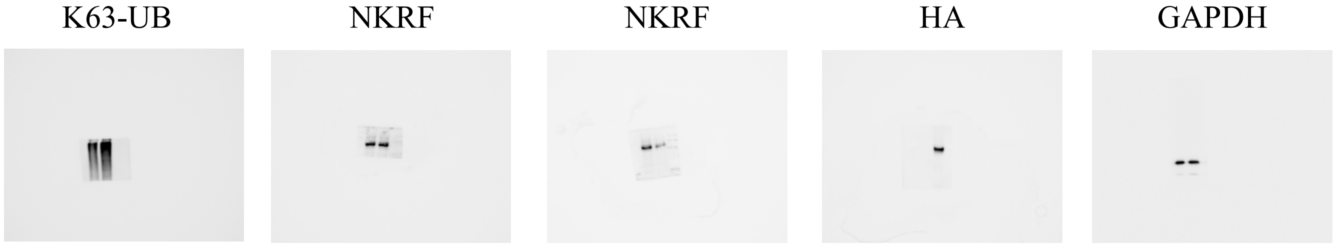

Figure 5

F

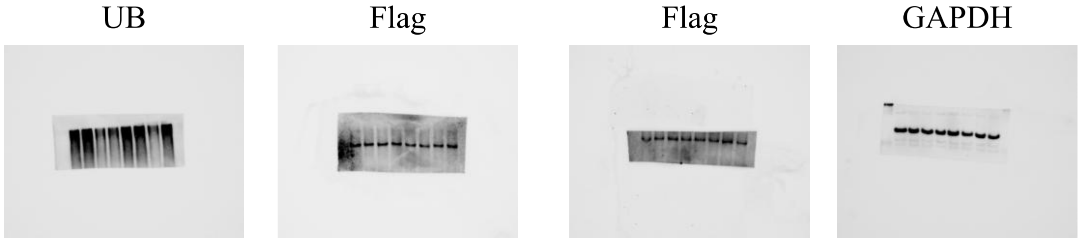

G

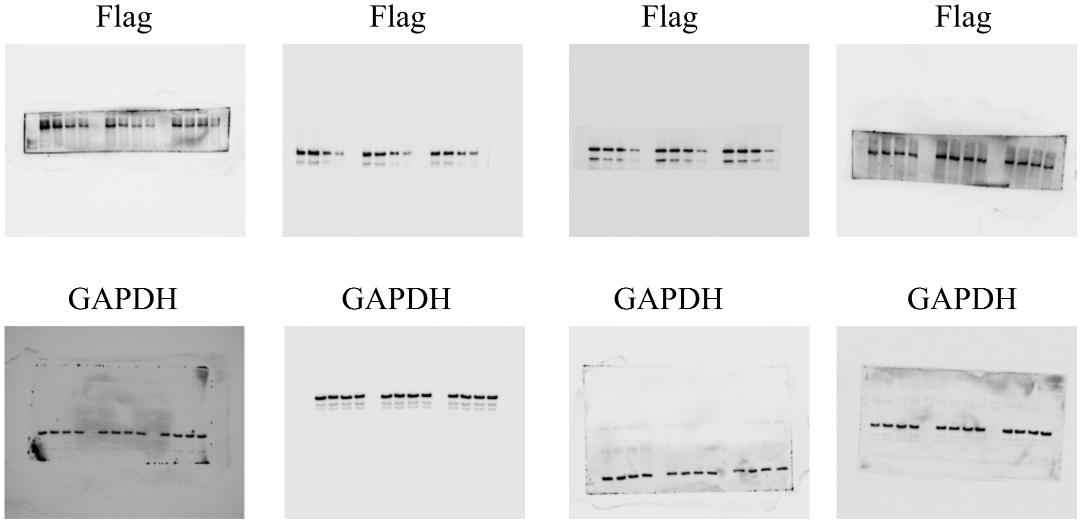

H

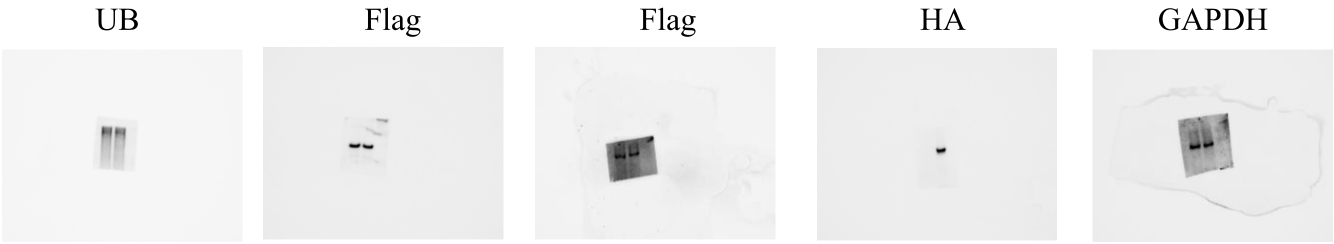

I

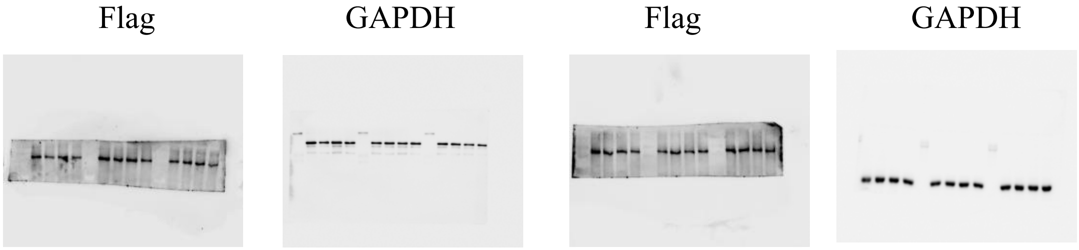

Figure 6

A

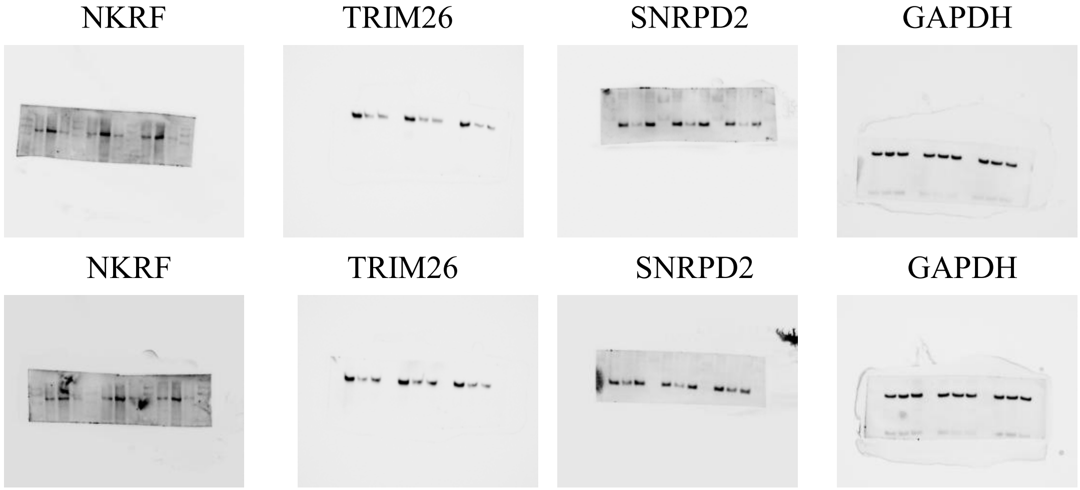

Figure 7

A

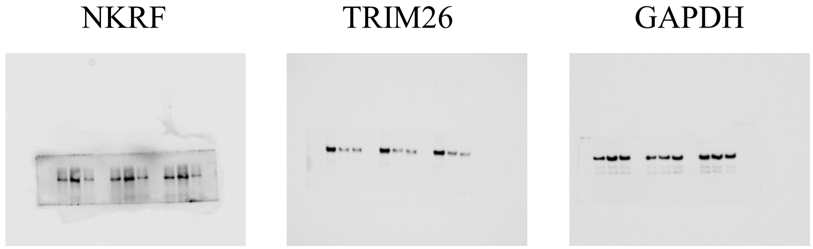

Supplement: Supplementary file 3 — Original Western Blots Data [file 41419_2026_8787_MOESM3_ESM.pdf]
